# Metabolic characterization of aggressive breast cancer cells exhibiting invasive phenotype: impact of non-cytotoxic doses of 2-DG on diminishing invasiveness

**DOI:** 10.1186/s12885-020-07414-y

**Published:** 2020-09-29

**Authors:** Mayumi Fujita, Kaori Imadome, Veena Somasundaram, Miki Kawanishi, Kumiko Karasawa, David A. Wink

**Affiliations:** 1 Department of Basic Medical Science for Radiation Damages, National Institute of Radiological Sciences, NIRS, National Institute for Quantum and Radiological Science and Technology, QST, 4-9-1, Anagawa, Inage-ku, Chiba-shi, Chiba-ken, Japan; 2 Department of Radiation Oncology, Tokyo Women’s Medical University, Tokyo, Japan; 3 Laboratory of Cancer Immunometabolism, Center for Cancer Research, National Cancer Institute, National Institutes of Health, Frederick, MD USA

**Keywords:** Breast cancer, Invasion, Metabolism, Glycolysis, TCA cycle, ETC, 2-DG

## Abstract

**Background:**

Metabolic reprogramming is being recognized as a fundamental hallmark of cancer, and efforts to identify drugs that can target cancer metabolism are underway. In this study, we used human breast cancer (BC) cell lines and established their invading phenotype (INV) collected from transwell inserts to compare metabolome differences and evaluate prognostic significance of the metabolome in aggressive BC invasiveness.

**Methods:**

The invasiveness of seven human BC cell lines were compared using the transwell invasion assay. Among these, INV was collected from SUM149, which exhibited the highest invasiveness. Levels of metabolites in INV were compared with those of whole cultured SUM149 cells (WCC) using CE-TOFMS. The impact of glycolysis in INV was determined by glucose uptake assay using fluorescent derivative of glucose (2-NBDG), and significance of glycolysis, or tricarboxylic acid cycle (TCA) and electron transport chain (ETC) in the invasive process were further determined in aggressive BC cell lines, SUM149, MDA-MB-231, HCC1937, using invasion assays in the presence or absence of inhibitors of glycolysis, TCA cycle or ETC.

**Results:**

SUM149 INV sub-population exhibited a persistent hyperinvasive phenotype. INV were hyper-glycolytic with increased glucose (2-NBDG) uptake; diminished glucose-6-phosphate (G6P) levels but elevated pyruvate and lactate, along with higher expression of phosphorylated-pyruvate dehydrogenase (pPDH) compared to WCC. Notably, inhibiting of glycolysis with lower doses of 2-DG (1 mM), non-cytotoxic to MDA-MB-231 and HCC1937, was effective in diminishing invasiveness of aggressive BC cell lines. In contrast, 3-Nitropropionic acid (3-NA), an inhibitor of succinate dehydrogenase, the enzyme that oxidizes succinate to fumarate in TCA cycle, and functions as complex II of ETC, had no significant effect on their invasiveness, although levels of TCA metabolites or detection of mitochondrial membrane potential with JC-1 staining, indicated that INV cells originally had functional TCA cycles and membrane potential.

**Conclusions:**

Hyper-glycolytic phenotype of invading cells caters to rapid energy production required for invasion while TCA cycle/ETC cater to cellular energy needs for sustenance in aggressive BC. Lower, non-cytotoxic doses of 2-DG can hamper invasion and can potentially be used as an adjuvant with other anti-cancer therapies without the usual side-effects associated with cytotoxic doses.

## Background

Breast cancer (BC) is a common cancer in women worldwide [1]. Although earlier diagnosis and improvements in treatment have reduced the mortality rate of BC, the incidence of BC is estimated to be increasing globally [1]. Thus, prevention and treatment of BC remain a major public health concern. BC subtypes, such as inflammatory breast cancer (IBC) and triple negative breast cancer (TNBC), are aggressive types of BC, which are extremely lethal and have higher potential for distant metastasis [2, 3]. Majority of BC patients succumb to metastasis. Thus, understanding the characteristics of the sub-population of cancer cells that exhibit the invasive phenotype is fundamental for discovering novel targets to block the invasion-metastasis cascade and ensure improved BC treatment.

Cancer cells adopt various strategies that allow them to be more aggressive such as changing their cellular metabolism [4]. Metabolic reprogramming is increasingly being recognized as a fundamental hallmark of cancer, and efforts to identify drugs that can target cancer metabolism are underway [5]. Several studies have revealed that oncogenes make cells more glycolytic [6, 7], and in fact, many tumor cells consume glucose and produce lactate at significantly higher rates than the surrounding tissue, even when enough oxygen exists [8, 9]. 2-deoxy-D-glucose (2-DG), a D-glucose mimetic, inhibits glycolysis due to formation and intracellular accumulation of 2-deoxy-D-glucose-6-phosphate, inhibiting the function of hexokinase and glucose-6-phosphate isomerase [10, 11]. 2-DG has a potential application as an adjuvant for improving cancer therapy, as it was to be able to reduce cancer cell viability [12–15] and has also been assessed in several clinical studies as an anticancer agent [16–18]. However, clinical use of 2-DG still has been carefully studied because of its side effects [11, 14]. Thus, combination of lower dose of 2-DG with other anticancer drugs, or with radiotherapy, is promising for clinical use [11]. A recent report showed that 2-DG is also effective in inhibiting migration and invasion ability of an invasive subclone of the TNBC cell line, Hs578T [19]. Due to limited therapeutic options for targeting metastasis, use of 2-DG for blocking cancer invasiveness is attractive. However, the study only showed the result of one BC cell line, Hs578T, and thus, further studies are required to clarify the role of glycolysis in BC invasion.

It was originally hypothesized that cancer cells utilize aerobic glycolysis because of mitochondrial respiratory dysfunction [20]. However, later evidence suggested that most tumor cells have functional tricarboxylic acid (TCA) cycle and electron transport chain (ETC), despite which, cancer cells favor the use of glucose to produce lactate rather than acetyl-CoA for TCA cycle [4, 20, 21]. Glutamine can be a primary source of citrate via reductive metabolism and is known to be used as a source of TCA metabolites in aggressive cancers [22, 23]. In addition, several reports have revealed that mitochondria, the site of TCA cycle and ETC, is an important organelle that destines a cell to a metastatic phenotype [24, 25]. Thus, it is still controversial which metabolic arm i.e., glycolysis or, TCA and ETC is significant for maintaining the invasive potential in BC.

Herein, we establish that BC invasive cells (INV) collected from transwell inserts is a discernible population with a persistent phenotype that are hyperinvasive. These cells showed upregulation of glucose uptake and were effectively targeted using 2-DG. This effectiveness of 2-DG on blocking invasion was observed in several aggressive BC cell lines, SUM149 (IBC), MDA-MB-231 (TNBC), and HCC1937 (BRCA1mut/TNBC), and of note, low dose of 2-DG (1 mM), non-toxic to MDA-MB-231 and HCC1937 viability, was effective in reducing their invasion. In contrast, blocking function of TCA cycle and ETC had no significant effect on their invasiveness, although levels of TCA metabolites or detection of mitochondrial membrane potential with JC-1 staining indicated that INV cells originally had functional TCA cycles and membrane potentials. Overall, our results convincingly establish that inhibition of glycolysis, such as with low dose of 2-DG, is a viable therapeutic option to blocking aggressive BC invasiveness.

## Methods

### Cell culture and reagents

Human BC cell lines, MCF-7 (ATCC® HTB-22), BT-474 (ATCC® HTB-20), SK-BR-3 (ATCC® HTB-30), MDA-MB-468 (ATCC® HTB-132), MDA-MB-231 (ATCC® HTB-26), HCC1937 (ATCC® CRL-2336) derived from different breast cancer subtypes were purchased from ATCC (Manassas, VA, USA), and SUM149 (Asterand SUM-149PT) from Asterand (Hertfordshire, UK) in 2013.The cell lines were authenticated by provider with morphology, karyotyping, and PCR based approaches. The subtypes of each cell lines were further authenticated with determining ER, PgR and HER2 expression in a recent paper [26]. Cells were cultured in complete RPMI (Roswell Park Memorial Institute) medium (Nakalai, Kyoto-shi, Kyoto-fu, Japan) consisting of 10% fetal bovine serum (FBS, HyClone Laboratories, GE Healthcare, Logan, UT, USA), 2 mM L-glutamine, and 100 U/ml penicillin/streptomycin (Gibco, Gaithersburg, MD, USA) at 37 °C and 5% CO_2_. Cells in logarithmic growth phase were seeded at an appropriate density, and used for all experiments. 2-deoxy-D-glucose (2-DG) (Sigma-Aldrich, St. Louis, MO, USA), 3-Nitropropionic Acid (3-NA) (Cayman Chemical, Ann Arbor, MI, USA), CB-839 (Cayman Chemical), Phloretin (Tokyo Chemical Industry, Nihonbashi, Tokyo, Japan) were the inhibitors used in this study.

### Transwell invasion assay

The invasive potential of seven BC cell lines were examined as previously described [27–29]. The transwell membrane was photographed under bright field using a BZ-9000 fluorescence microscope (Keyence, Osaka, Japan) using a Nikon Plan Apo 4x/0.2 lens. Numbers of invaded cells in each field were counted with the particle counting application of ImageJ software (Version 1.52q) [27].

For inhibitor studies, cells were pre-treated with 2-DG, 3-NA, or CB-839 for 16 h, trypsinized, suspended in serum-free RPMI with appropriate inhibitor, and then used for the invasion assay. Inhibitor was also added to the lower well, and the invasion assay was performed for 24 h. In addition, surviving fraction of cells treated with each inhibitor for 16 h was examined by counting viable cells using trypan blue staining.

### INV preparation and re-invasion assay

To prepare the invaded cells of SUM149 (INV), transwell invasion assays were performed as described previously [28, 29]. For the re-invasion assay, WCC and INV were both cultured for 1, 4, 7, 12, or 19 days, and the transwell invasion assay was performed repeatedly with these cells as described previously [29].

### Sample preparation and metabolome analysis by CE-TOFMS

INV and WCC (1 × 10^5 cells/sample for INV, and 1 × 10^6 cells/sample for WCC, respectively) were used for the extraction of intracellular metabolites [28], and the metabolome analysis was performed with CE-TOFMS as described earlier [28, 30–32]. The collection methods of INV and WCC for the metabolome analysis were summarized in reference [28]. Analysis of raw data measured by CE-TOFMS was performed as described previously [28, 33].

### Immunoblotting

Immunoblotting was performed as described previously [34]. Briefly, cells were lysed in 2× Laemmli sample buffer, followed by electrophoresis using the Novex® NuPAGE® SDS-PAGE Gel system (ThermoFisher Scientific, Waltham, MA, USA). Primary antibodies against Pyruvate Dehydrogenase E1-alpha subunit (9H9AF5) (PDH), phosphorylated-Pyruvate Dehydrogenase E1-alpha subunit Ser300 (pPDH S300), Ser232 (pPDH S232), or Ser239 (pPDH S239) (Abcam, Cambridge, UK) were used along with horseradish peroxidase-conjugated anti-mouse IgG or anti-rabbit IgG (Amersham Biosciences; Buckinghamshire, UK). Bands were detected by enhanced chemiluminescence and visualized with a Lumino image analyzer, LAS 4000 (Fujifilm, Tokyo, Japan) using the ImageQuant LAS 4000 Control Software.

### Flow cytometry

In order to examine the glucose uptake into living cells, 2-NBDG (2-Deoxy-2-[(7-nitro-2,1,3-benzoxadiazol-4-yl)amino]-D-glucose, Cayman Chemical), a fluorescent derivative of glucose, was used. For WCC, cells were separately cultured as 3 groups; control, 2-NBDG treating, or 2-NBDG + Phloretin (inhibitor for the glucose transporter) treating groups. On the day of flow cytometry experiment, cells were washed with PBS and medium were changed to fresh RPMI supplemented with 0.5% FBS with or without 100 μM Phloretin. After 4 h incubation, 60 μM 2-NBDG was added to 2-NBDG group, and 2-NBDG + Phloretin group, and were incubated another 1 h. Cells were then washed with PBS, incubated with Accutase for 15 min (Innovative Cell Technologies Inc.), collected and used for flow cytometry analysis in accordance with the manufacturer’s instructions (CytoFLEX S with Analysis Software; Beckman Coulter). For INV, invasion assay was performed 1 day before the flow cytometry experiment. After 24 h INV cells underneath the transwell were directly treated with 2-NBDG or 2-NBDG + Phloretin. WCC were also subjected to same treatment groups. Cells were collected with Accutase for 15 min and used for the flow cytometry analysis.

### Immunofluorescence study and image acquisition

Immunofluorescence study, and image acquisition were performed with some modifications from previous studies [27]. Briefly, cells were cultured on glass slide chamber with phenol red free RPMI at appropriate density. On the day of immunofluorescence experiment, cells were washed with PBS, and medium was changed to fresh phenol red free RPMI with or without 3-NA. After 5 h incubation at 37 °C/5% CO_2_, 5, 5′, 6, 6′-tetrachloro-1, 1′, tetraethylbenzimidazolocarbocyanine iodide (JC-1, mitochondrial membrane potential detection indicator, ThermoFisher Scientific) and NucBlue® Live ReadyProbes (Nuclear staining solution, ThermoFisher Scientific) were added to each well, and incubated for 30 min. Cells were washed with PBS and fresh phenol red free RPMI was added, and images were acquired using a BZ-9000 fluorescence microscope (Keyence, Osaka, Japan) using a 10X PlanFluor NA 0.30 Ph1 lens with BZ filters for TRITC, GFP-B, and DAPI. Images were uniformly processed in Creative Cloud Photoshop CC using the brightness and contrast tools.

### Lactate measurement

Cells were separately cultured as 3 groups; control, 0.3 mM 2-DG treatment, or 1 mM 2-DG treatment groups. On the day before the experiment, cells were washed with PBS and medium was changed to fresh RPMI with appropriate concentration of 2-DG. After 16 h incubation, conditioned medium was collected and used for the assay according to the manufacture’s protocol (Lactate Assay Kit-WST, Dojindo).

### Spheroid invasion assay

Spheroid invasion assay was performed in ultra-low attachment multiple 96 well plates (Sigma-Aldrich) [28, 29]. Cells (5 × 10^3) were plated in each well and incubated in 37 °C CO_2_ incubator. After 48 h, spheroids were stained with JC-1 (1:50 concentration) for 30 min, embedded into phenol red-free collagen solution (custom version 3D Ready Atelocollagen, KOKEN CO., LTD., Bunkyo-ku, Tokyo, Japan), followed by 1 h incubation at 37 °C/5% CO_2_ to allow solidification of collagen solution. Image of spheroid was captured at 1 h and 24 h after embedding into collagen gel and fluorescence was detected and photographed with a BZ-9000 fluorescence microscope using a 4X PlanApo λ NA 0.20 lends with BZ filters for TRITC, GFP-B (Keyence, Osaka, Japan). Representative images were uniformly processed in Adobe Photoshop using the brightness and contrast tools.

### Statistical analysis

All results are shown as the mean +/− SD. Significance was analyzed using unpaired Student’s t-test. *P* value < 0.05 was considered significant.

## Results

### SUM149 showed the highest, persistent invasiveness among seven human BC cell lines

**Fig. 1:**
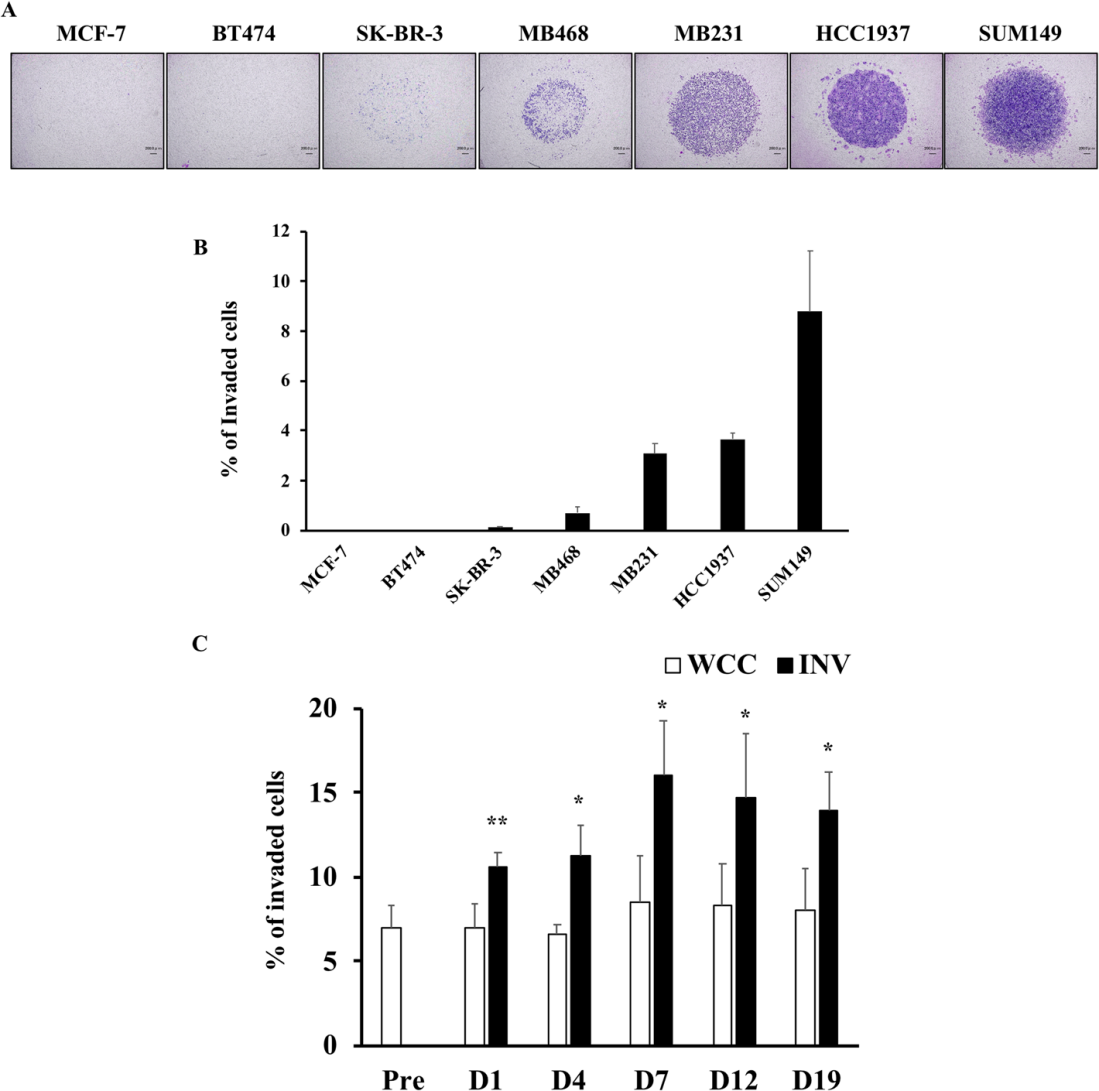
SUM149 was selected for collecting invaded cells from transwell inserts. **a** Invasion assay was performed with using seven human breast cancer cell lines, MCF-7, BT-474, SK-BR-3, MDA-MB-468, MDA-MB-231, HCC1937, and SUM149. Representative images of invaded cells reached underneath of the transwell membrane are shown. Scale bar: 200 μm. **b** Percent of invaded cells of seven human breast cancer cell lines is shown in graph. Data are presented as mean ± SDs of triplicate samples. **c** Whole cultured SUM149 cells (WCC) and INV collected from underneath of transwell membranes were used for invasion assay. Number of invaded cells were counted, and ratio of invaded cells of INV group to WCC group was summarized in graph. Data are presented as mean ± SDs of triplicate samples. *p < 0.05, **p < 0.01

To investigate the role of glycolysis, or TCA cycle and ETC in the invasive ability of BC cells, we first planned to establish BC invaded cells collected from transwell inserts (INV) as we previously reported with human pancreatic cancer cells [29]. Number of cells which successfully invade through the Matrigel, vary depending on the cell line used for the invasion assay; some cell lines show no invading cells, while other cell lines have an evident proportion of invading cells. In order to prepare BC INV, we first had to choose the appropriate cell line, with a good number of invading cells, for collecting INV from transwell inserts. Thus, we used seven human BC cell lines to compare their invasiveness. Cell lines that originated from aggressive BC such as MDA-MB468, MDA-MB-231, HCC1937 (TNBC cell lines), or SUM 149 (IBC cell line) exhibited intermediate to high invasiveness, with 0.70% ± 0.25, 3.07% ± 0.41, 3.66% ± 0.26, and 8.80% ± 2.41 invaded cells respectively. In contrast, less aggressive BC cell lines such as MCF-7 (Luminal-HER2 negative type), BT-474 (Luminal-HER2 positive type), or SK-BR-3 (HER2 positive type) showed minimal to no invasiveness (0.00, 0.00%, or 0.14% ± 0.02, respectively) (Fig. 1a-b). Among the seven cell lines, SUM149 was selected for collecting INV, because it exhibited the highest number of invaded cells. To examine whether SUM149 INV had higher invasive phenotype compared to whole cultured SUM149 (WCC), a re-invasion assay was performed. Day1 collected INV from the undersurface of transwell membranes showed 1.65 ± 0.11 times higher invasiveness compared to WCC. This increased invasiveness of INV was sustained until Day 19; being 1.71 ± 0.27 times higher on Day 4, 1.89 ± 0.47 times higher on Day 7, 1.77 ± 0.46 times higher on Day 12, and 1.73 ± 0.29 times higher on Day 19 (Fig. 1c), indicating that INV had persistently higher invasiveness compared to WCC. Thus, we used INV and WCC from SUM149 for further study, comparing the levels of glycolysis and TCA cycle metabolites.

### SUM149 invaded cells showed hyper-glycolytic phenotype compared to WCC

**Fig. 2:**
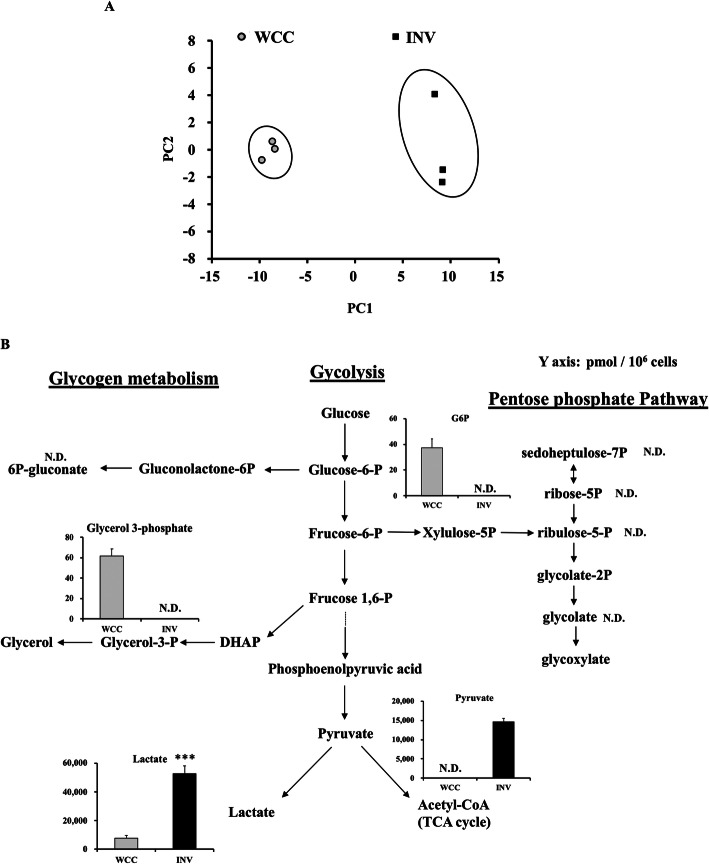
SUM149 INV cells showed hyper-glycolytic phenotype compared to WCC. **a** Principal component analysis of metabolites detected in WCC versus INV was performed and is shown in the graph (*n* = 3). **b** Metabolomics profile of WCC and INV were analyzed by CE-TOFMS. The concentrations of glycolytic intermediates measured in WCC or INV are shown in graph. Y axis represents the metabolite concentration in pmol / 10^6 cells. Data are presented as mean ± SD of samples (*n* = 3). ***p < 0.001 vs. WCC., N.D.: Not detected

SUM149 INV and SUM149 WCC were collected, and metabolites in these cells were analyzed by CE-TOFMS (Supplemental Table 1). PCA indicated that the metabolomic profile of INV was very distinct from that of WCC (Fig. 2a). Importantly, the glycolytic intermediate, glucose-6-phosphate (G6P), was below detection limit in INV, whereas the downstream metabolites, pyruvate and lactate were both increased compared to those of WCC, suggesting that INV metabolized glucose into lactate more efficiently/ faster than WCC did (Fig. 2b). Also, a portion of the G6P in WCC is used up in the production of glycerol-3-phosphate (G3P).

**Fig. 3:**
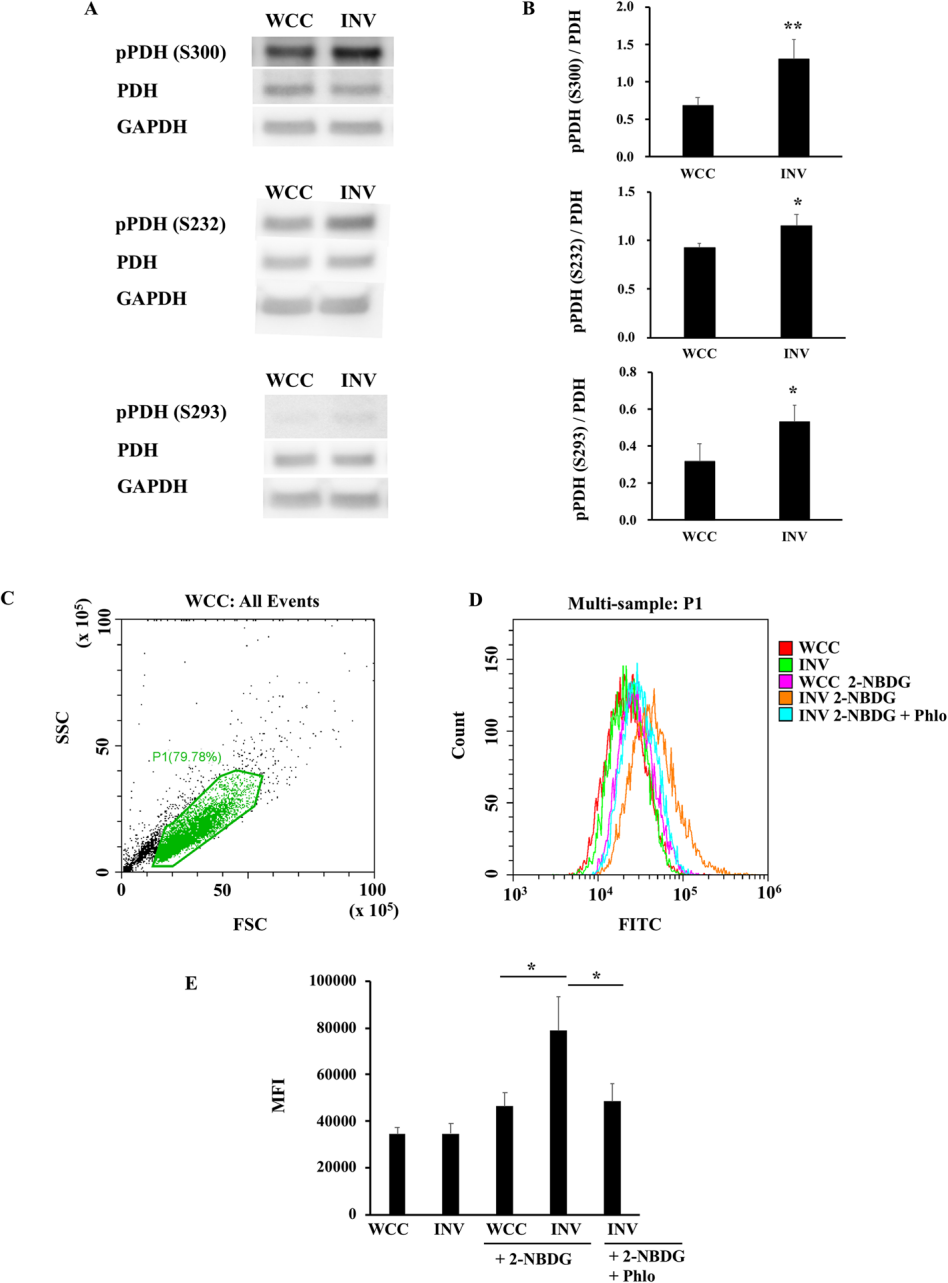
Phosphorylation of PDH was increased in SUM149 INV compared to **WCC** Expression of pPDH S300, pPDH S232, pPDH S239, PDH, and GAPDH proteins of INV and WCC were examined with western blot analysis and bands were shown in (**a**), and the graphs of phosphorylated-PDH vs. pan-PDH were shown in (**b**). Data are presented as mean ± SD of samples (*n* = 3). *P < 0.05, **P < 0.01 vs. WCC. Uncropped full-length blots images were shown in Supplemental Figure 1. **c** Glucose uptake into WCC or INV was examined with 2-NBDG, and analyzed with flow cytometer. Live cells were gated as P1 (**c**), and levels of FITC and mean fluorescent intensity (MFI) was shown in graph (**d**), and (**e**), respectively. 2-NBDG represents a fluorescent tracer used for monitoring glucose uptake, and Phlo represents phloretin, inhibitor for the glucose transporter, respectively. Data are presented as mean ± SD of samples (*n* = 3). **p < 0.01, ***p < 0.001

Pyruvate dehydrogenase (PDH) is a component enzyme of the pyruvate dehydrogenase complex, which catalyzes conversion of pyruvate to acetyl-CoA, the entry substrate for the TCA cycle, rather than converting pyruvate into lactate [35]. De-phosphorylation of specific serine residues of the E1-alpha subunit of PDH, Ser300 (pPDH S300), Ser232 (pPDH S232), or Ser239 (pPDH S239), activates the conversion of pyruvate to acetyl CoA, whereas phosphorylation inactivates the enzyme, resulting in increased conversion into lactate. We found that the expression of pPDH S300, pPDH S232, and pPDH S239 were all significantly increased in INV compared to WCC (Fig. 3a-b, Supplemental Figure 1A-C), suggesting that PDH enzyme activity was reduced in INV, consistent with increased production of lactate (Fig. 2b). In addition, uptake of 2-NBDG, a fluorescent tracer used for monitoring glucose uptake into live cells, was higher in INV compared to WCC, and this was reduced by the addition of phloretin, a natural phenol which inhibits a variety of transporters including sodium/D-glucose cotransporter, SGLT1, which is known as the sodium-dependent glucose transporter [36] (Fig. 3c-e). Overall, these data indicated that INV had impaired PDH activity and consumed higher amount of glucose and metabolized it into lactate than WCC did.

### SUM149 invaded cells showed functional TCA cycle and ETC system but these pathways do not contribute to BC invasion

**Fig. 4:**
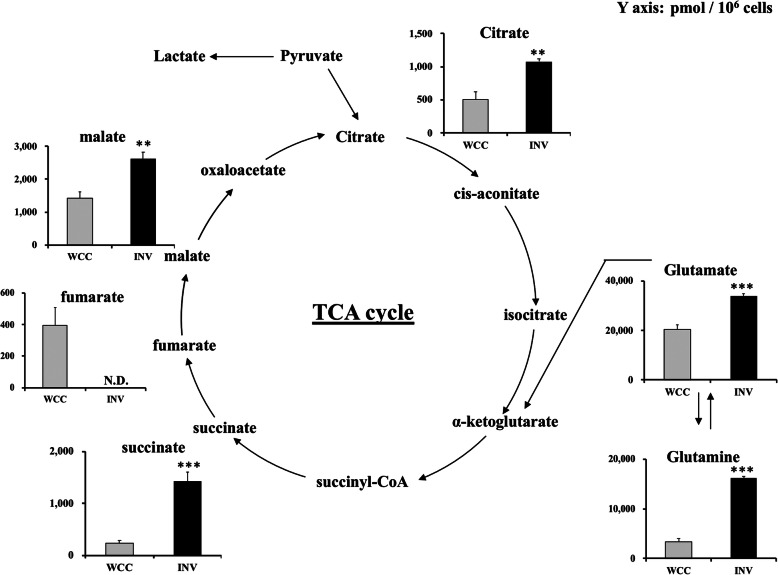
Comparison of TCA cycle metabolites of SUM149 WCC vs INV. TCA cycle metabolites of WCC and INV were analyzed by CE-TOFMS, and concentrations were shown in graph. Y axis represents the metabolite concentration in pmol / 10^6 cells. Data are presented as mean ± SD of samples (*n* = 3). **p < 0.01, ***p < 0.001 vs. WCC., N.D.: Not detected

Despite the higher rate of glycolysis and reduced conversion of glucose into acetyl-CoA in INV, the level of TCA cycle metabolites, such as citrate, succinate, and malate, were still higher in INV than WCC (Fig. 4), indicating that TCA cycle was functional in INV and was potentially being fueled by other sources. Additionally, to examine ETC in INV, we stained 2-day old SUM149 spheroids with JC-1, a mitochondrial membrane potential (ΔΨm) indicator. Red-stained cells (high ΔΨm) appear to be moving outward from the spheroid and invading the collagen gel, and green-stained cells (low ΔΨm) tend to stay within/ near the spheroid (Supplemental Figure 2), suggesting that SUM149 invading cells possess active mitochondria with high membrane potential. High mitochondrial membrane potential is generated by the reductive transfer of electrons through ETC protein complexes I–IV, which provides the energy to drive protons against their concentration gradient across the inner mitochondrial membrane [37]. Thus, SUM149 INV cells, which exhibited the active mitochondria, would also have a functional ETC system.

**Fig. 5:**
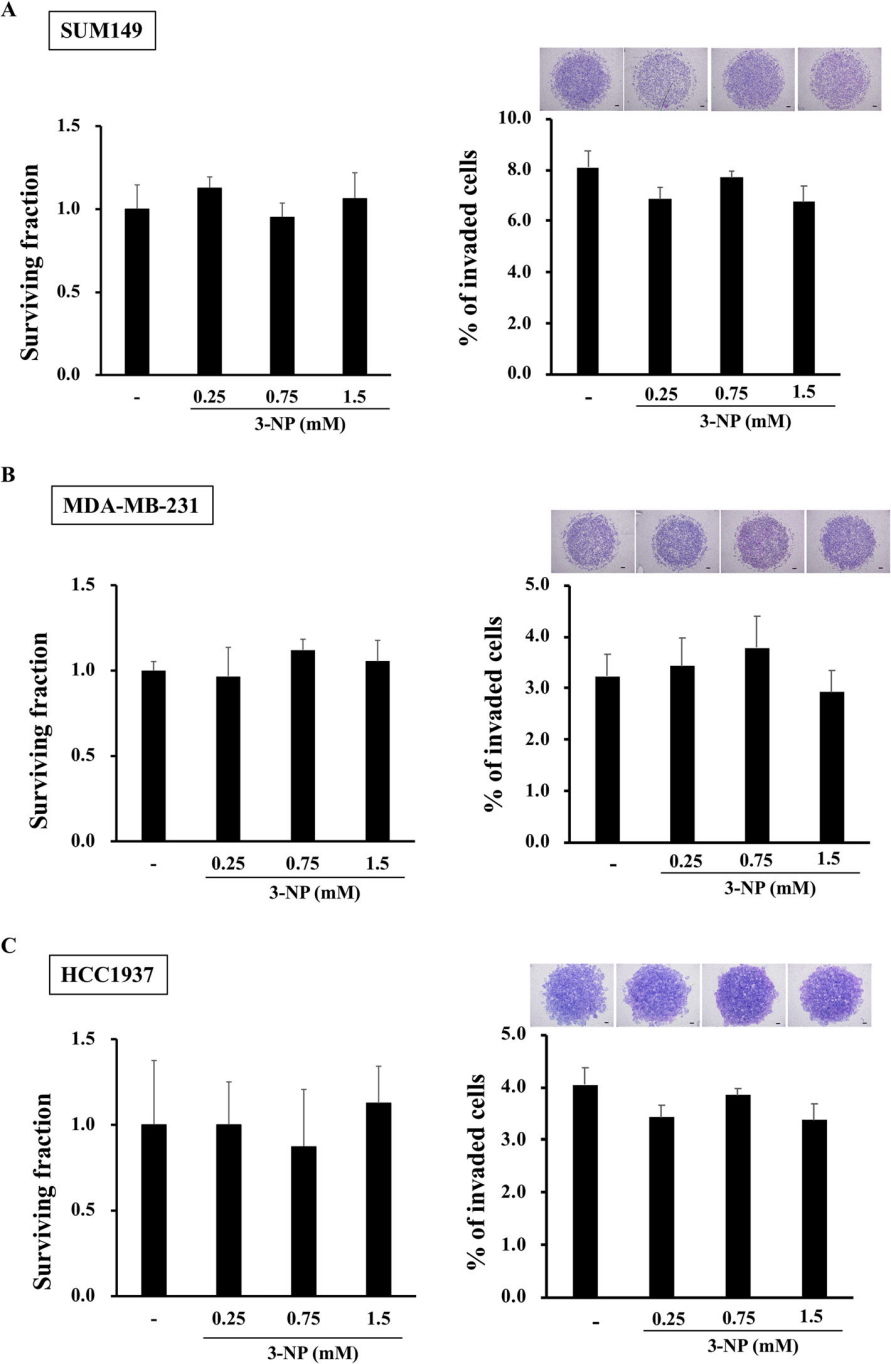
Inhibitor for TCA cycle and ETC had no significant effects on BC invasiveness Cells were treated with 0.25, 0.75, or 1.5 mM 3-NA for 16 h, and surviving fraction, and invasiveness were studied. Ratio of surviving vs. control (no 3-NA treatment), and percent of invaded cells with representative image of invaded cells reached underneath of the transwell membrane were shown for SUM149 (**a**), MDA-MB-231 (**b**), and HCC1937 (**c**), respectively. Data are presented as mean ± SDs of triplicate samples. Scale bar: 200 μm

To investigate the role of TCA cycle and ETC in BC invasiveness, we next performed invasion assay with 3-NA, an inhibitor of succinate dehydrogenase, the enzyme that oxidizes succinate to fumarate in the TCA cycle, and functions as complex II of the ETC [38]. Hence, 3-NA simultaneously inhibits TCA cycle and ETC. Interestingly, use of 3-NA had no significant effects on invasiveness of SUM149, MDA-MB-231, or HCC1937 (Fig. 5a-c), although their mitochondrial membrane potential was reduced with 3-NA treatment (Supplemental Figure 3A-C), suggesting that TCA cycle and ETC are less important for their invasive capability. In addition, since INV seemed to produce lactate rather than acetyl-CoA, glutamine from media or produced by anaplerotic reactions may be the source of fuel for TCA cycle via reductive metabolism to α-ketoglutarate [22, 23], as glutamine and glutamic acid levels were also higher in INV compared to WCC (Fig. 4, Supplemental Figure 4A-B). Thus, we next performed SUM149 invasion assay using glutamine-free medium, or Glutaminase 1 (GLS1) inhibitor, CB-839. However, use of Glutamine free medium or CB-839 had no significant effects on reducing their invasion (Supplemental Figure 5A-B). Overall, these data suggested that TCA cycle and ETC system are less significant for BC invasion.

### Glycolysis inhibitor diminished BC invasion

**Fig. 6:**
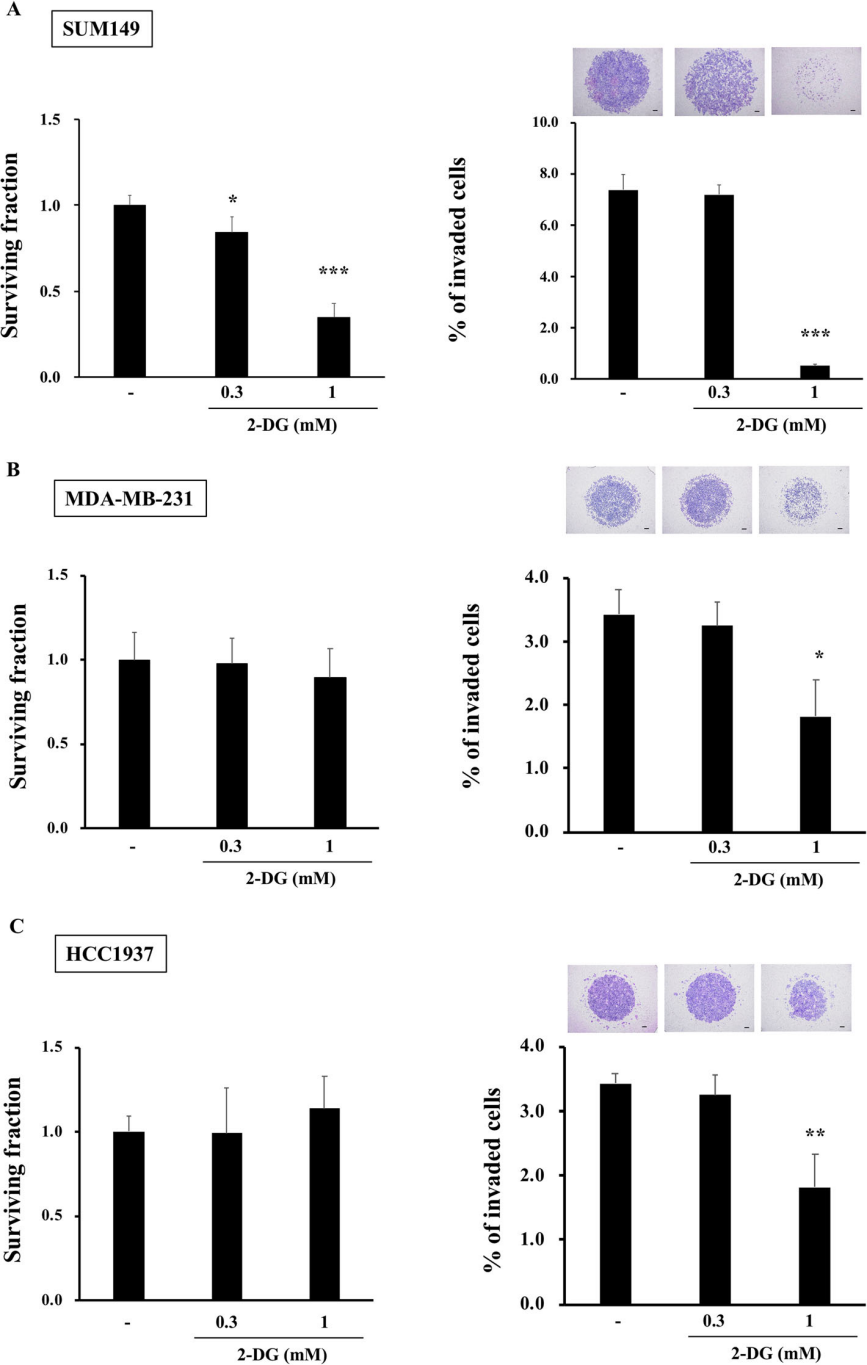
Low dose of glycolysis inhibitor, 2-DG, diminished BC invasion Cells were treated with 0.3 or 1 mM 2-DG for 16 h, and surviving fraction, and invasiveness were determined. Ratio of surviving vs. control (no 2-DG treatment), and percent of invaded cells with representative image were shown for SUM149 (**a**), MDA-MB-231 (**b**), and HCC1937 (**c**), respectively. Data are presented as mean ± SDs of triplicate samples. *p < 0.05, **p < 0.01, ***p < 0.001. Scale bar: 200 μm

In order to examine the role of glycolysis in BC invasion, we next used glycolysis inhibitor, 2-DG, for the invasion assay. First, we delineated the 2-DG dose requirement for inhibition of glycolysis versus cellular toxicity. If we used the level of 2-DG which blocks cell viability, then we could not examine the effect of de-activated glycolysis on the invasiveness because the cells had already succumbed. Thus, we chose lower 2-DG concentrations, 0.3 and 1 mM, compared to higher concentrations used in other studies focusing on the effect of 2-DG on blocking cell viability; Cheng et al. showed 5 mM 2-DG was effective in reducing viability of pancreatic cancer cells, AsPC-1 and PANC-1 [39], Lucantoni et al. reported 10 mM 2-DG was effective in reducing the number of colonies of BC cell lines, MCF-7 and HDQ-PI [40], and Valera et al. reported that 5 mM 2-DG was non-toxic for bladder cancer cell line, but could effectively sensitize these cancer cells to other anti-cancer drugs [41]. We first confirmed that treatment of cells with 0.3 or 1 mM 2-DG for 16 h reduced lactate production by SUM149, MDA-MB-231, and HCC1937 cell lines (Supplemental Figure 6A-B), suggesting that low concentration of 2-DG was sufficient to reduce glycolysis. Inhibition of glycolysis is known to reduce cell viability [12–15]. Thus, we next investigated the sensitivity of SUM149, MDA-MB-231, or HCC1937 to same concentrations of 2-DG (Fig. 6a-c). Adding 0.3 or 1 mM 2-DG to the culture media for 16 h, had no significant effects on reducing surviving fraction in MDA-MB-231 or HCC1937, but importantly, 1 mM 2-DG significantly reduced the invasiveness of these cells (Fig. 6b-c). In the case of SUM149, 1 mM 2-DG was already toxic, and hence reduced both surviving fraction and the invasion (Fig. 6a), but the level of reduction was much more drastic in invasion, compared to those observed in surviving fraction. In conclusion, data indicated that glycolysis has important role in aggressive BC invasiveness, and lower dose of 2-DG, non-toxic to cell viability, was efficient to reduce BC cell invasiveness.

## Discussion

Most cancer cells rely on glycolysis even when oxygen is available, a phenomenon known as the Warburg effect [42]. Aggressive type of BC, viz. IBC and TNBC, have been shown to possess higher levels of glycolytic activity than ER+ breast cancer cells [43–46], however, the role of glycolysis specifically on their invasiveness is still unclear. In this study, we establish that INV from an IBC cell line had a persistently hyper-invasive phenotype. Such INV showed upregulation of glucose uptake, and were effectively targeted using a glycolysis inhibitor, 2-DG. Several earlier studies have shown the ability of 2-DG to block cancer growth [11–15, 47]. However, many of the clinical trials using 2-DG were discontinued due to the adverse side effects caused by the high doses of 2-DG [11, 18]. Importantly, the concentration of 2-DG that we used in this study, 1 mM, is five times lower than the doses used in recent studies focusing on blocking cancer cell growth (≥5 mM) [39–41], and 1 mM was shown as non-toxic on cell survival [41]. In accordance, we observed that 1 mM of 2-DG did not reduce cell survival of MDA-MB-231 and HCC1937 cell lines, but of note, it was significantly effective in blocking their invasion. Sadhbh O’Neill et al have reported that 2-DG is effective to inhibit migration and invasion ability of invasive BC subclone, Hs578T, but they used much higher concentration of 2-DG i.e., 15 mM (15 times the dose used in the current study), and have not focused on the effect of 2-DG with lower dose [19]. The associated side effects of such a high dose of 2-DG would impede its use in clinical trials. However, lower doses of 2-DG which can still block cancer invasiveness, as we have shown here in aggressive BC cell lines, could be effectively applied as adjuvants to other anti-cancer therapies.

Several reports have shown that cancer cells have functional TCA cycle and ETC [4, 20, 21], concordantly, we observed that invading cells that move outward from spheroids embedded in collagen gel exhibited high mitochondrial membrane potential, suggesting that they possessed functional ETC. Blocking TCA and ETC with pharmacological inhibitor treatment diminished mitochondrial membrane potential, but it had no significant effects on blocking their invasion, indicating that TCA and ETC were less important than glycolysis to drive BC invasiveness. Glutamine can be a primary source fueling the TCA cycle [22, 23]. A recent study has shown that GLS inhibitor was effective in diminishing cell growth by limiting influx of glutamine derivatives into the TCA cycle, but this effect was only detected in the cells which have high levels of GLS, suggesting that the role of glutamine for TCA cycle is cell line-specific [48]. In this study, we used glutamine free media or GLS1 inhibitor (CB-839) to investigate the role of glutamine-TCA cycle pathway in SUM149. However, the no significant change was observed on their invasiveness. As expected from this data and previous literature, expression of GLS was low in SUM149 cells compared to other BC cells (data not shown) hence, inhibition of GLS1 had no effect in these cells. Also, the INV cells produce higher levels of all non-essential amino acids compared to WCC cells (Supplemental Figure 4) and could potentially fuel TCA through anaplerosis even in the absence of glutamine in the media [49].

Several studies have revealed that metformin is effective in blocking growth of aggressive cancer, including TNBC [50, 51]. Metformin has been used to treat type II diabetes, and its function is the inhibition of mitochondrial membrane complex I [51]. Blocking of mitochondrial membrane complex I decreases proton-driven synthesis of ATP, causing cellular energetic stress and activation of AMPK, which in turn impairs cell proliferation [51]. Thus, mitochondrial respiration system may be important for the cancer cell growth, rather than for the invasiveness. In addition, it is well known that many of the amino acids are synthesized from TCA cycle intermediates [49], which were important source for cell living or may be for invasion. In this study, we found that most of the amino acid levels were significantly increased in INV compared to WCC (Supplemental Figure 4). Inhibiting mitochondrial respiration complex II with 0.75 mM or 1.5 mM 3-NA for 16 h did not reduce survival of SUM149, MDA-MB-231, and HCC1937 in this study. However, in the case of MDA-MB-468, the least aggressive of the three TNBC cell lines, interestingly, treatment with the same doses of 3-NA, for 16 h drastically diminished cell growth and we were unable to collect the cells to use for the further invasion assay (data not shown). Thus, mitochondrial respiration system or TCA cycle may have significant role in cancer growth on certain cell types. The role of TCA cycle or ETC in SUM149, MDA-MB-231, and HCC1937 will be further delineated in future studies. However, it is now clear that although the TCA is not critical to the invasive process, they also have an active TCA cycle (through anaplerosis) which provides for cellular energy needs for sustenance, while glycolysis caters to the need for fast energy production required for invasion to occur within the nutrient deprived tumor conditions.

Epithelial mesenchymal transition (EMT) confers metastatic properties to cancer cells by increasing mobility, invasion, and metastasis [52, 53]. Interestingly, SNAI1, the transcription factor that represses E-cadherin expression (the marker of EMT induction), also enhances gene expression patterns that promote glucose uptake and glycolysis [54]. Although we have not determined the expression of E-cadherin in SUM149 INV, it is possible, given the higher invasiveness and higher glycolytic rate, that SUM149 INV was also in the mesenchymal state, similar to our previous report about INV established from human pancreatic cell line, PANC-1, that showed higher ability than WCC, to invade and metastasize in mice, exhibited reduced E-cadherin expression with induction of pro-metastatic genes [29].

## Conclusion

Intra-tumor heterogeneity can regulate cancer progression, resistance to therapy, and relapse [55, 56]. Development of effective therapeutic strategies that target the invasion-metastasis cascade depends on an in-depth understanding of the fundamental differences between cancer cells that exhibit the invasive phenotype versus the non-invasive phenotype. In this study, we established that the SUM149 INV sub-population exhibited a persistent phenotype that was hyperinvasive, and of note, we have clearly demonstrated that inhibiting glycolysis with lower, non-cytotoxic doses of 2-DG was effective in diminishing invasiveness of aggressive BC cell lines. Appropriate combinations of tumoricidal agents such as ionizing radiation and chemotherapeutic drugs with low dose of 2-DG can potentially provide unique opportunities to selectively destroy tumors and block invasion-metastasis cascade, and are expected to reduce toxicity to normal tissues and significantly enhance the therapeutic efficacy in aggressive BC such as IBC and TNBC.

## Supplementary information

**Additional file 1: Supplemental Figure 1.** Full-length blot images used for Figure 3.

**Additional file 2: Supplemental Figure 2.** Mitochondrial membrane potential of SUM149 invading cells.

**Additional file 3: Supplemental Figure 3.** Effects of 3-NP on mitochondrial membrane potential.

**Additional file 4: Supplemental Figure 4.** Levels of amino acids in WCC and INV.

**Additional file 5: Supplemental Figure 5.** Glutamine had no significant effects on SUM149 invasiveness.

**Additional file 6: Supplemental Figure 6.** Lactate measurement.

**Additional file 7: Table 1.** List of metabolites measured in WCC or INV of SUM149.

## Data Availability

Not applicable.

## Abbreviations

2-DG: 2-deoxy-D-glucose
2-NBDG: 2-Deoxy-2-[(7-nitro-2,1,3-benzoxadiazol-4-yl)amino]-D-glucose
3-NA: 3-Nitropropionic acid
BC: Breast cancer
CE-TOFMS: Capillary electrophoresis-time-of-flight mass spectrometry
EMT: Epithelial-to-mesenchymal transition
ETC: Electron transport chain
FBS: Fetal bovine serum
G6P: Glucose-6-phosphate
G3P: Glycerol-3-phosphate
GLS1: Glutaminase 1
IBC: Inflammatory breast cancer
INV: Invaded cells
MFI: Mean fluorescent intensity
PCA: Principal component analysis
PDH: Pyruvate dehydrogenase
pPDH: Phosphorylated-pyruvate dehydrogenase
pPDH S232: Phosphor-Pyruvate Dehydrogenase E1-alpha subunit Ser232
pPDH S239: Phosphor-Pyruvate Dehydrogenase E1-alpha subunit Ser239
pPDH S300: Phosphor-Pyruvate Dehydrogenase E1-alpha subunit Ser300
RPMI: Roswell Park Memorial Institute
SGLT: Sodium/D-glucose cotransporter
TCA cycle: Tricarboxylic acid cycle
TNBC: Triple negative breast cancer
WCC: Whole cultured cells
