# Supplementary figures and images for: Metabolic characterization of aggressive breast cancer cells exhibiting invasive phenotype: impact of non-cytotoxic doses of 2-DG on diminishing invasiveness
Source: BMC Cancer. 2020 Sep 29;20:929. doi: 10.1186/s12885-020-07414-y (PMC7525976; doi:10.1186/s12885-020-07414-y)

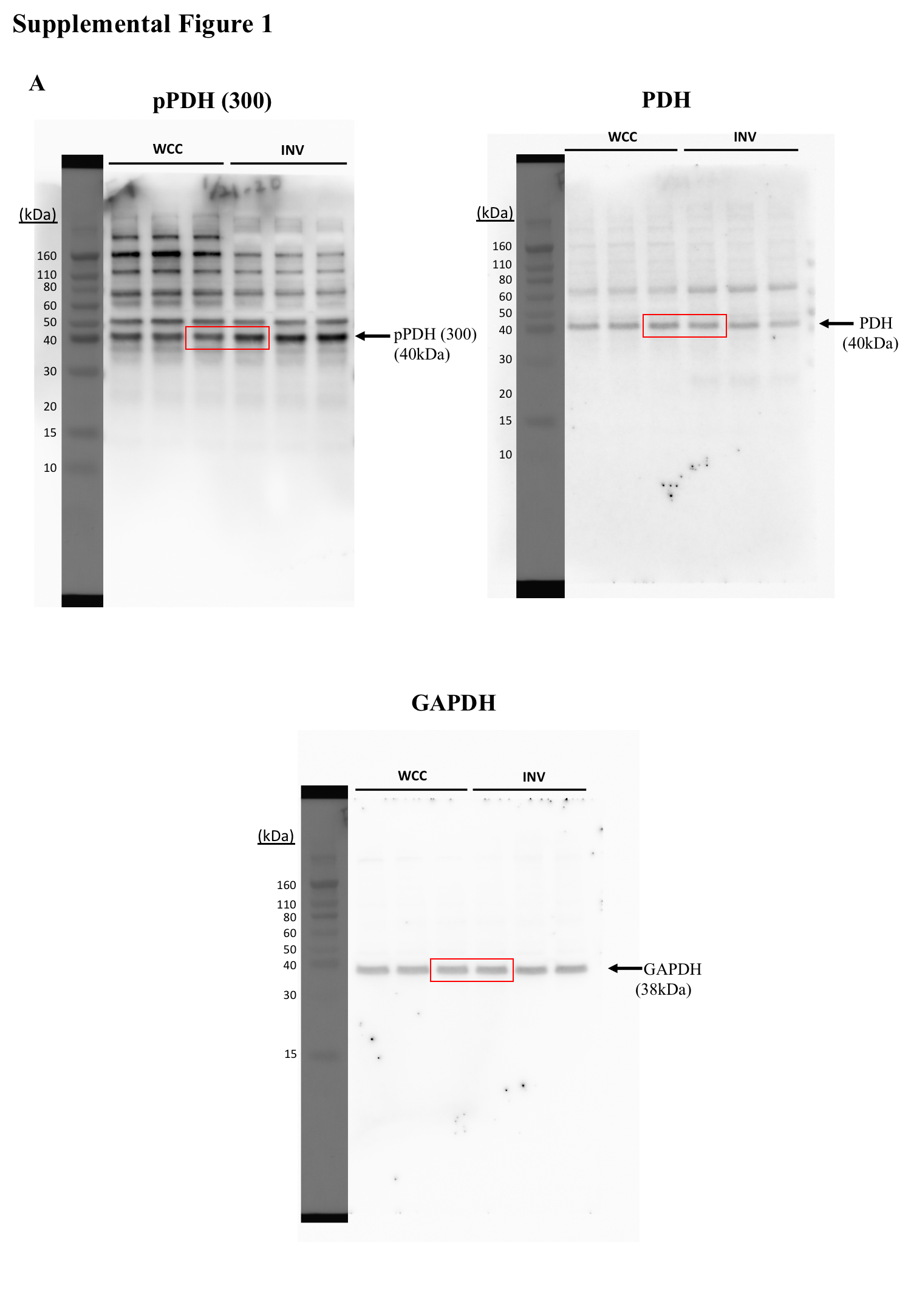

Supplement: Supplementary file 1 — Additional file 1: Supplemental Figure 1. Full-length blot images used for Figure 3. [file 12885_2020_7414_MOESM1_ESM.zip › Supplemental Figure 1AR2.tiff]

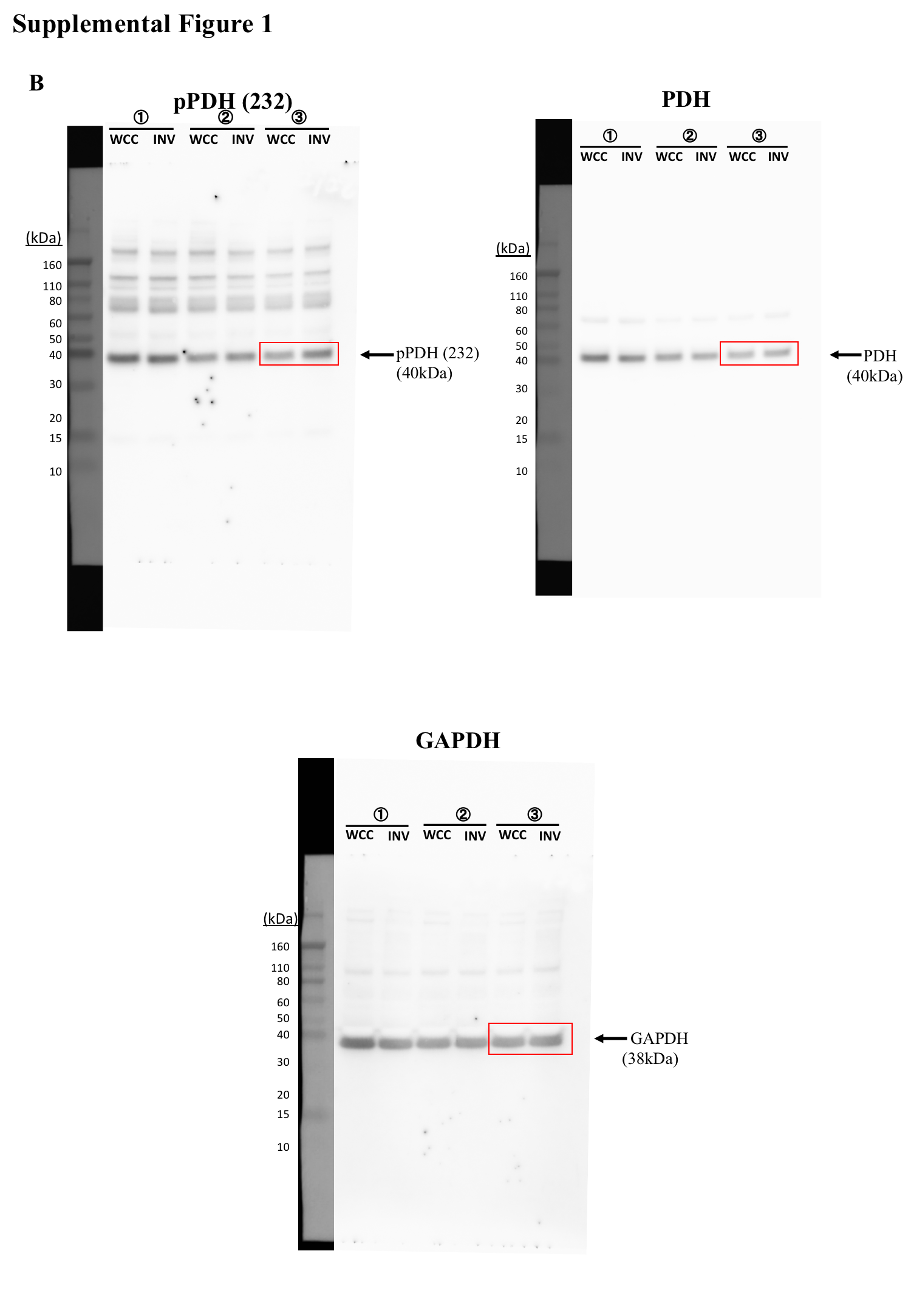

Supplement: Supplementary file 1 — Additional file 1: Supplemental Figure 1. Full-length blot images used for Figure 3. [file 12885_2020_7414_MOESM1_ESM.zip › Supplemental Figure 1BR2.tiff]

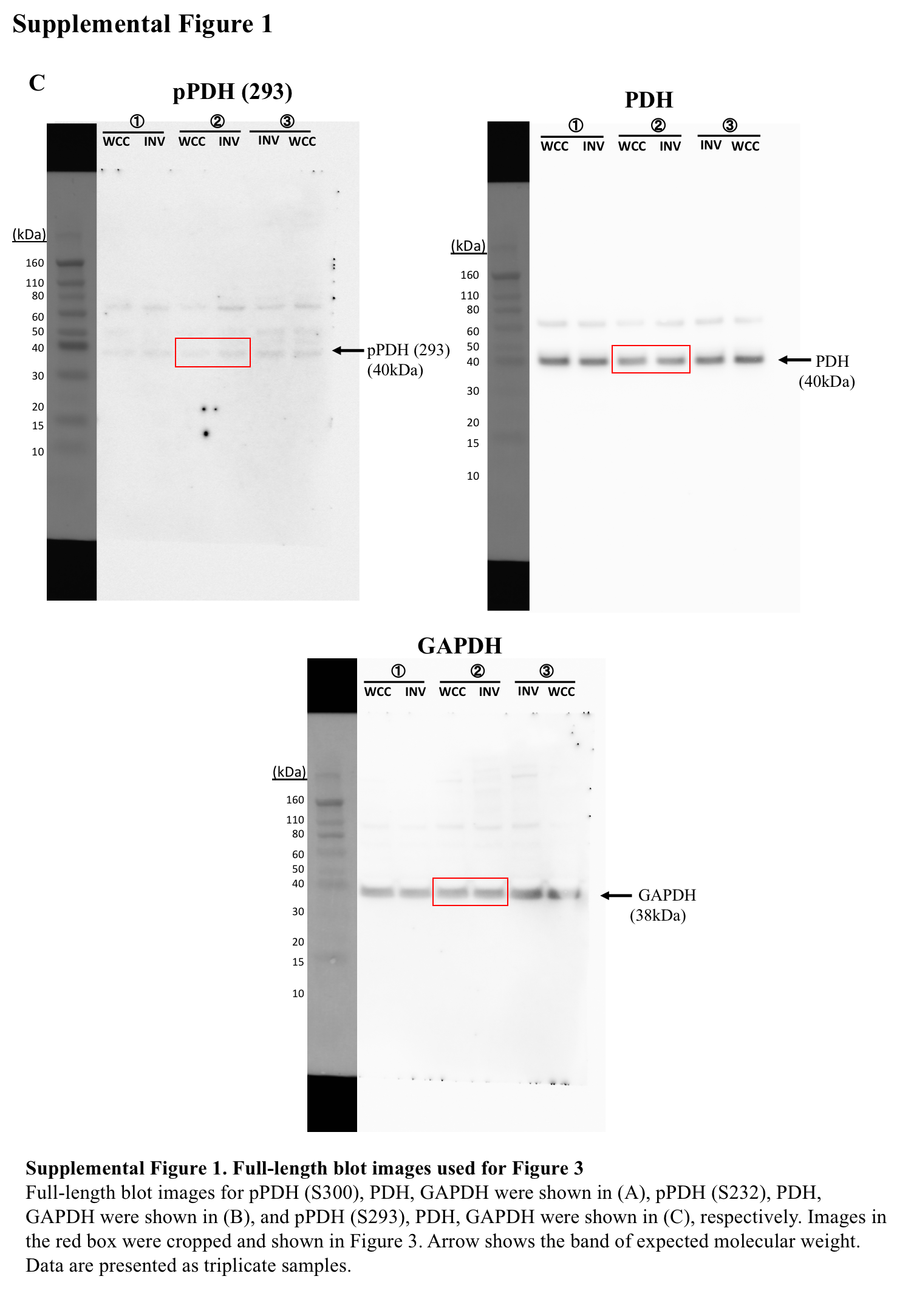

Supplement: Supplementary file 1 — Additional file 1: Supplemental Figure 1. Full-length blot images used for Figure 3. [file 12885_2020_7414_MOESM1_ESM.zip › Supplemental Figure 1CR2.tiff]

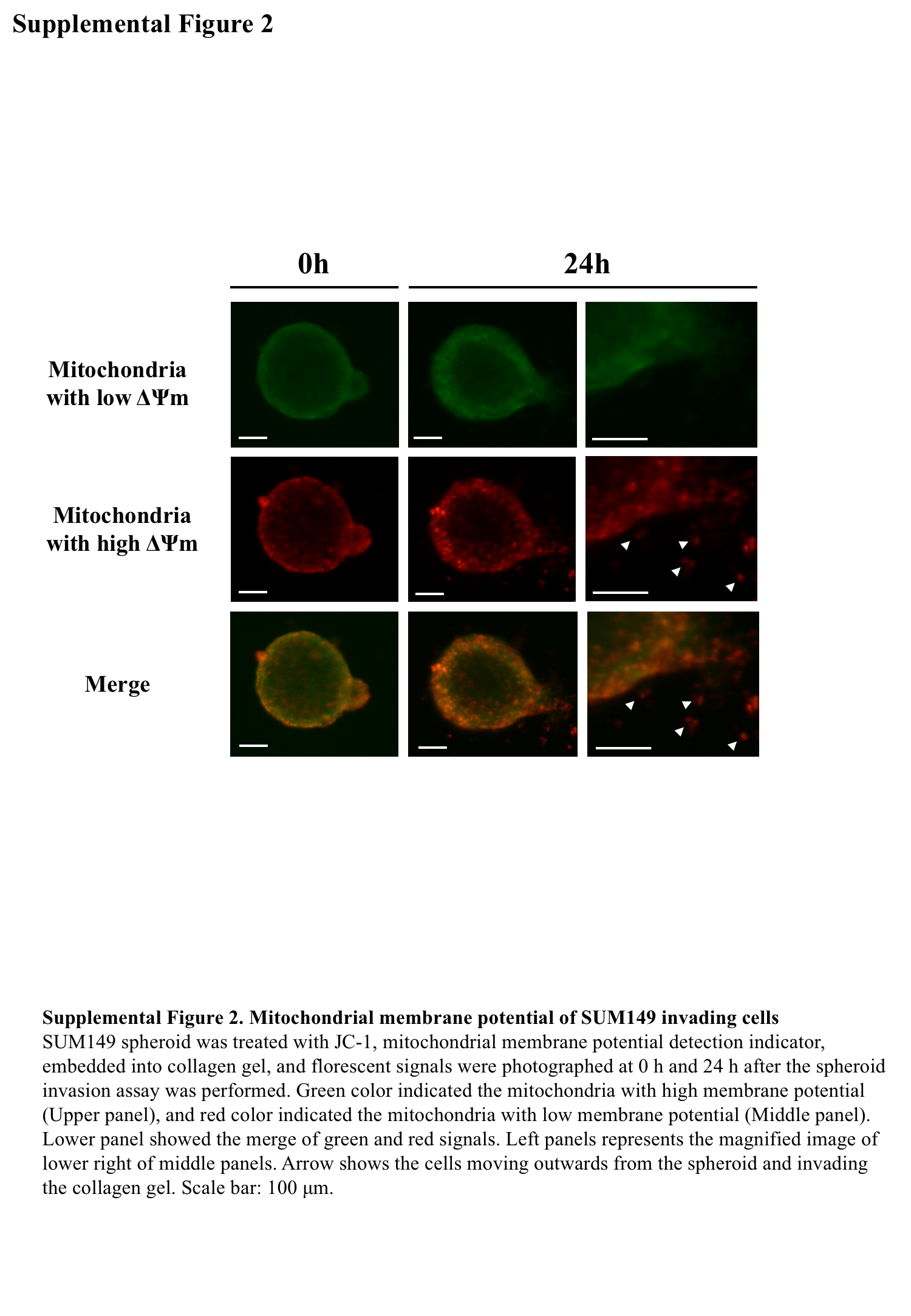

Supplement: Supplementary file 2 — Additional file 2: Supplemental Figure 2. Mitochondrial membrane potential of SUM149 invading cells. [file 12885_2020_7414_MOESM2_ESM.tiff]

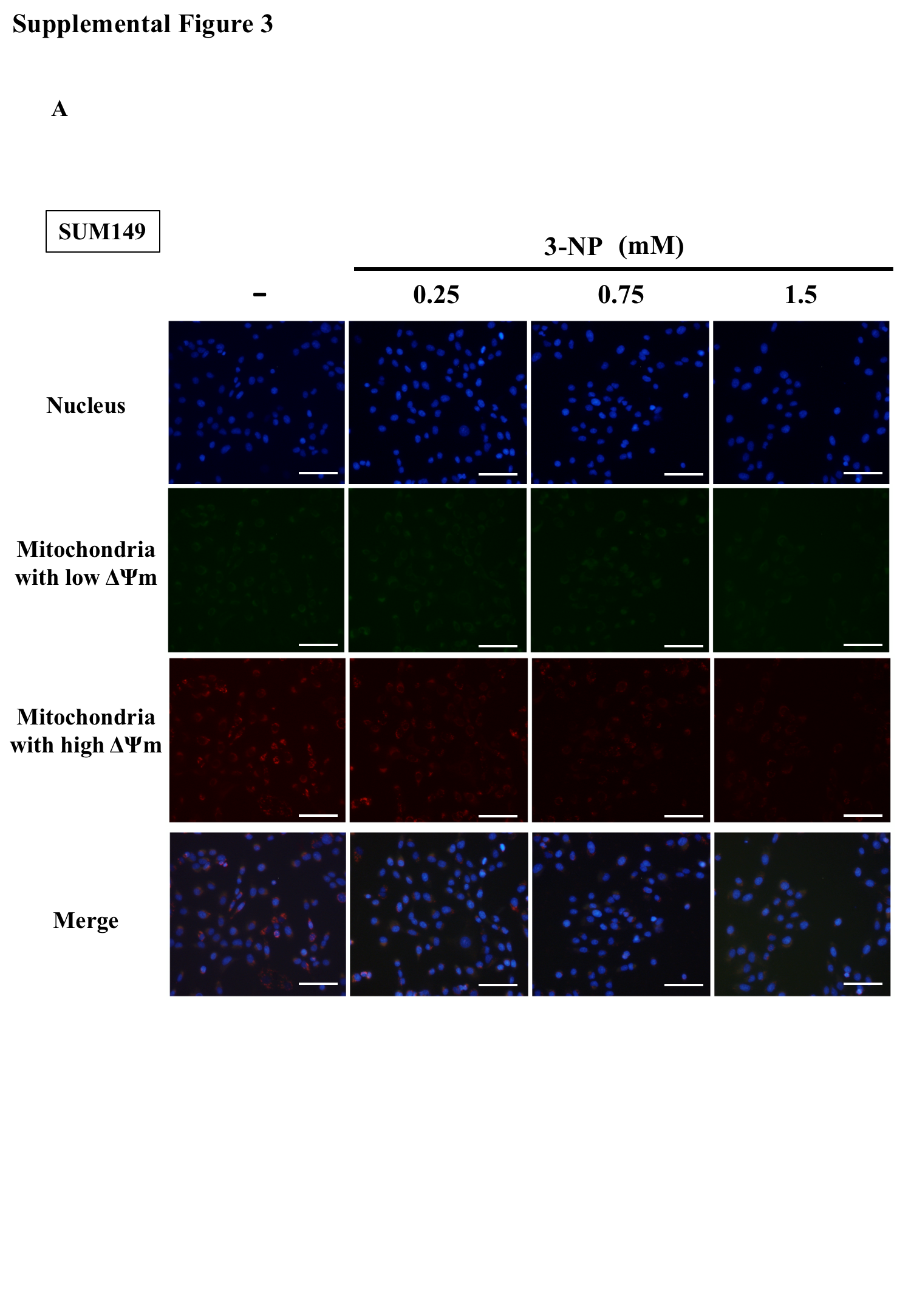

Supplement: Supplementary file 3 — Additional file 3: Supplemental Figure 3. Effects of 3-NP on mitochondrial membrane potential. [file 12885_2020_7414_MOESM3_ESM.zip › Supplemental Figure 3AR2.tiff]

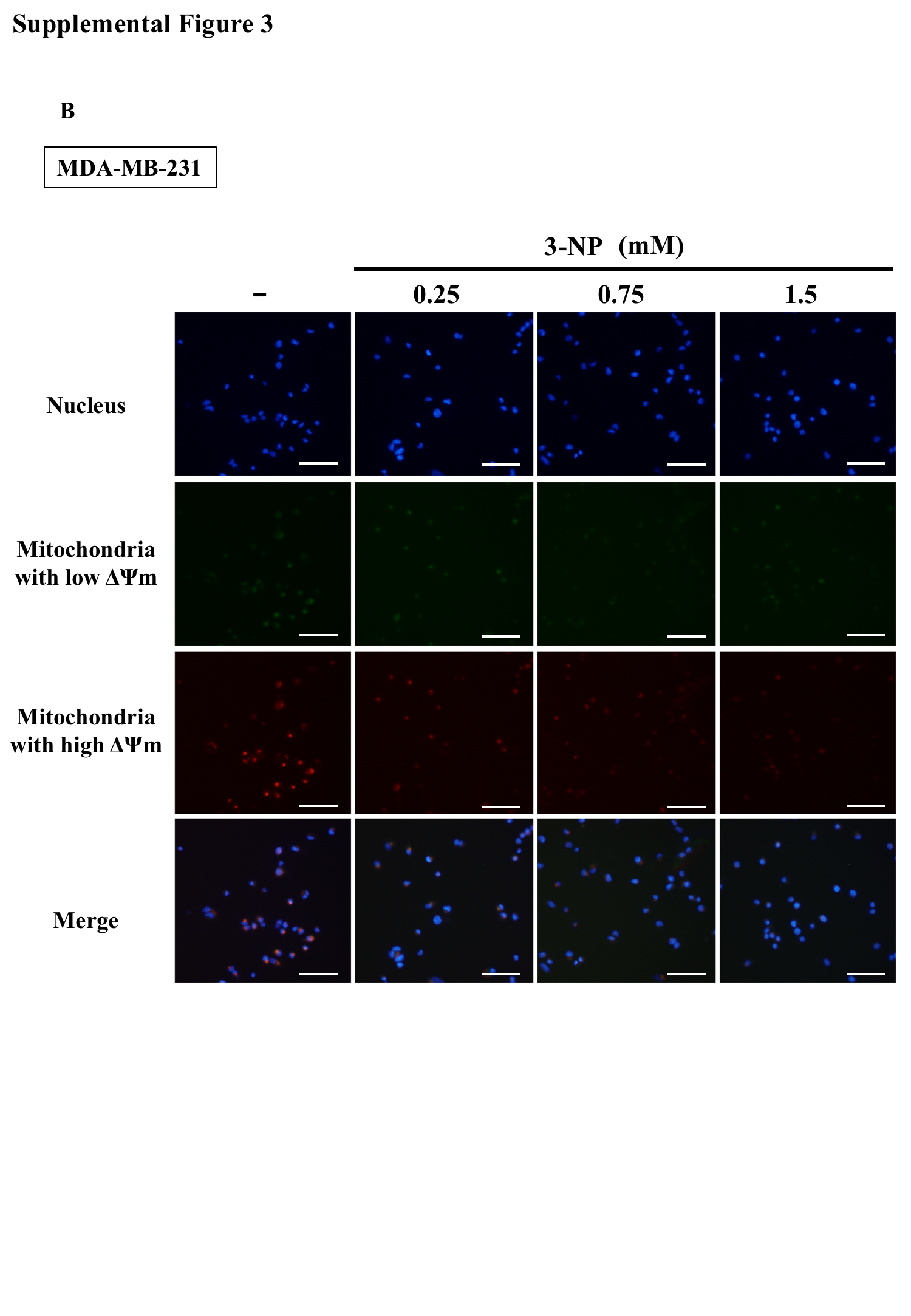

Supplement: Supplementary file 3 — Additional file 3: Supplemental Figure 3. Effects of 3-NP on mitochondrial membrane potential. [file 12885_2020_7414_MOESM3_ESM.zip › Supplemental Figure 3BR2.tiff]

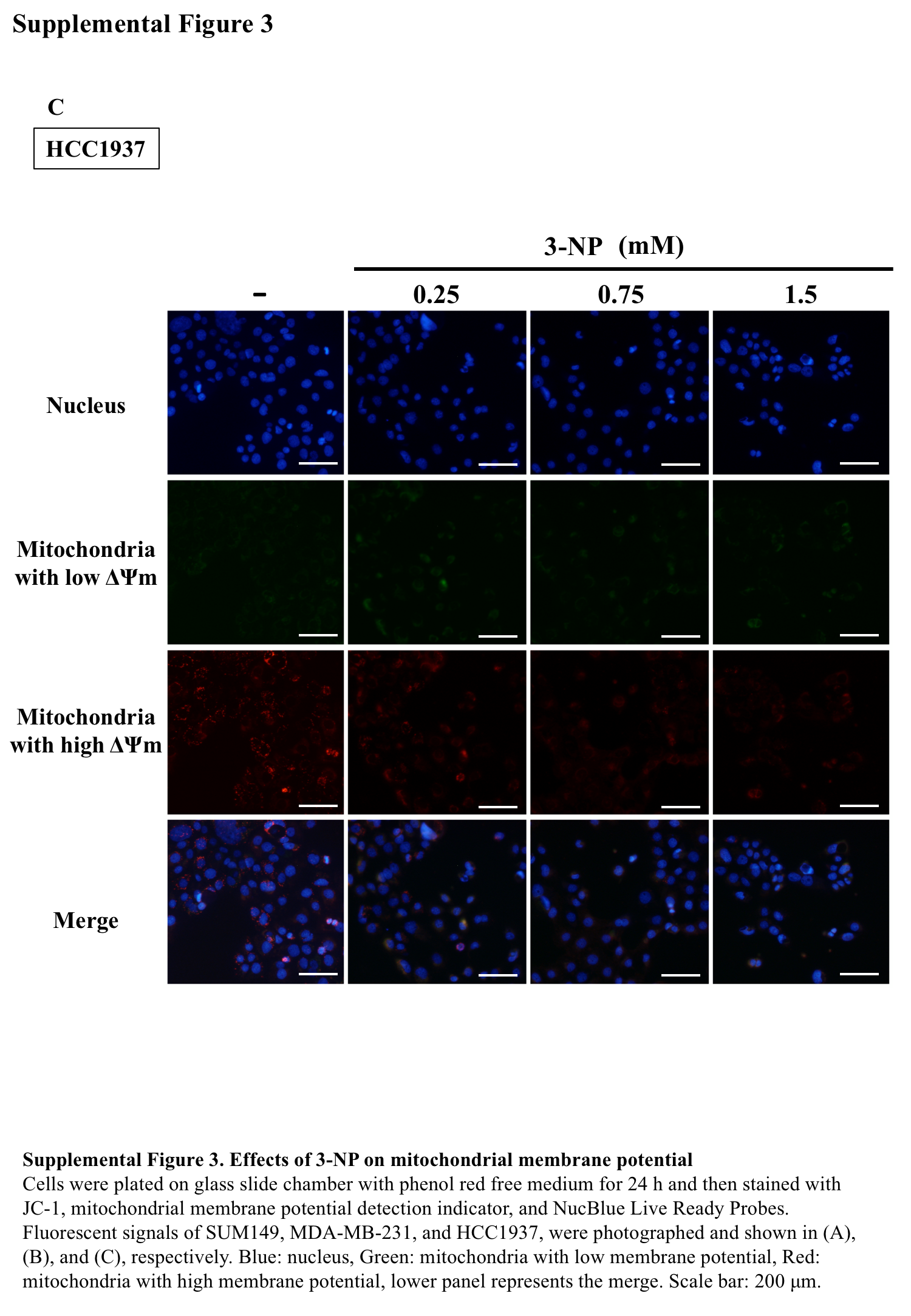

Supplement: Supplementary file 3 — Additional file 3: Supplemental Figure 3. Effects of 3-NP on mitochondrial membrane potential. [file 12885_2020_7414_MOESM3_ESM.zip › Supplemental Figure 3CR2.tiff]

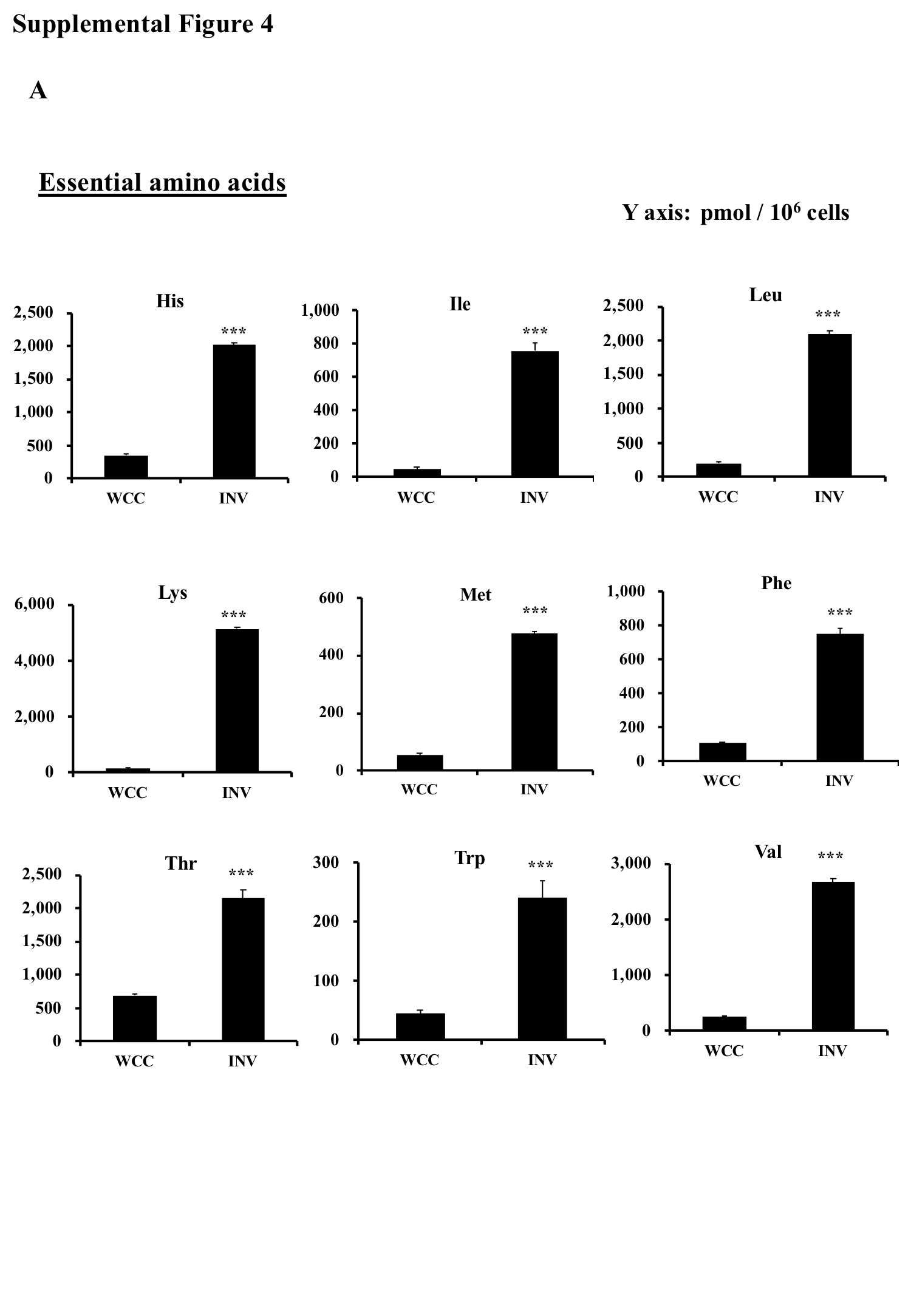

Supplement: Supplementary file 4 — Additional file 4: Supplemental Figure 4. Levels of amino acids in WCC and INV. [file 12885_2020_7414_MOESM4_ESM.zip › Supplemental Figure 4AR2.tiff]

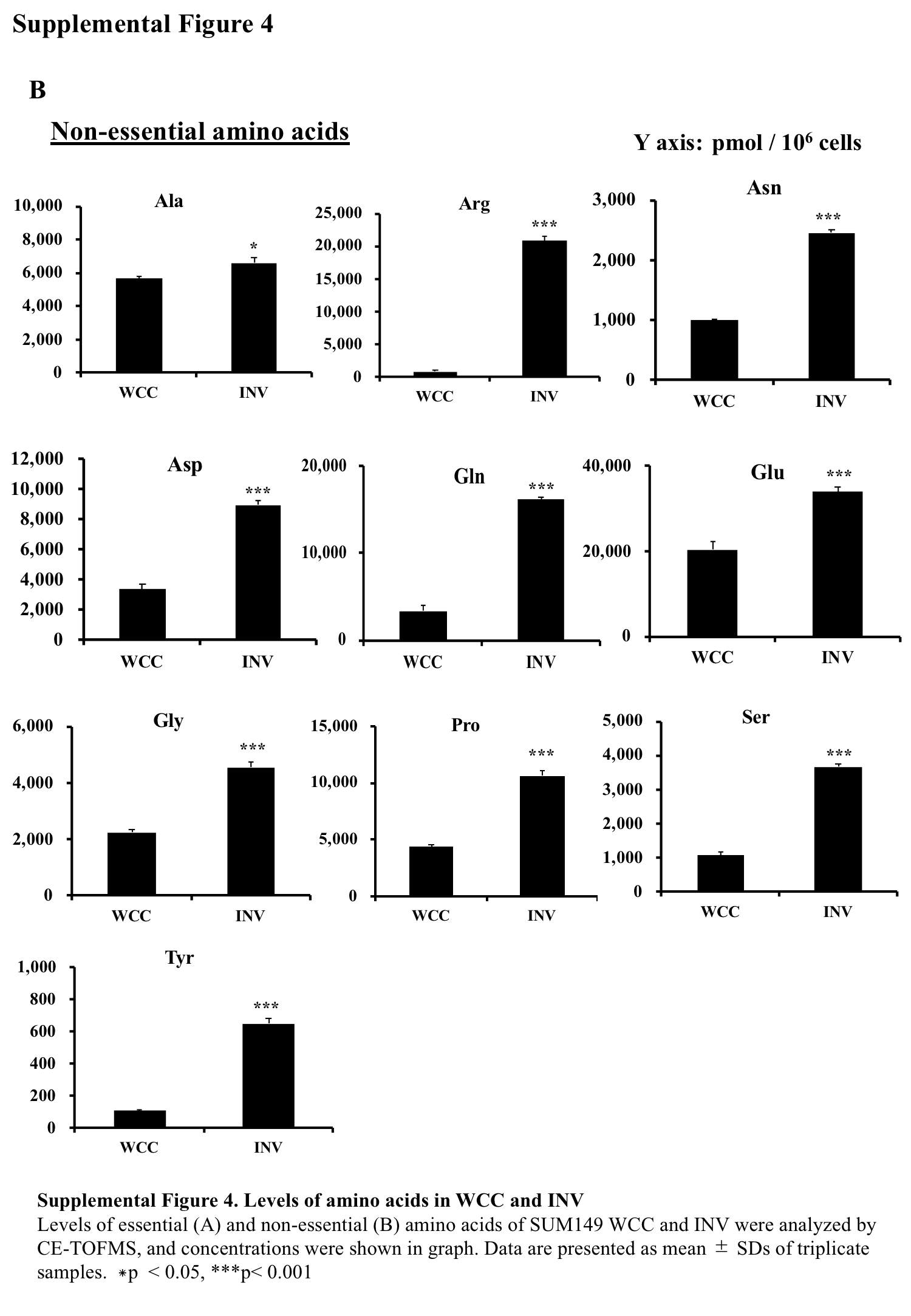

Supplement: Supplementary file 4 — Additional file 4: Supplemental Figure 4. Levels of amino acids in WCC and INV. [file 12885_2020_7414_MOESM4_ESM.zip › Supplemental Figure 4BR2.tiff]

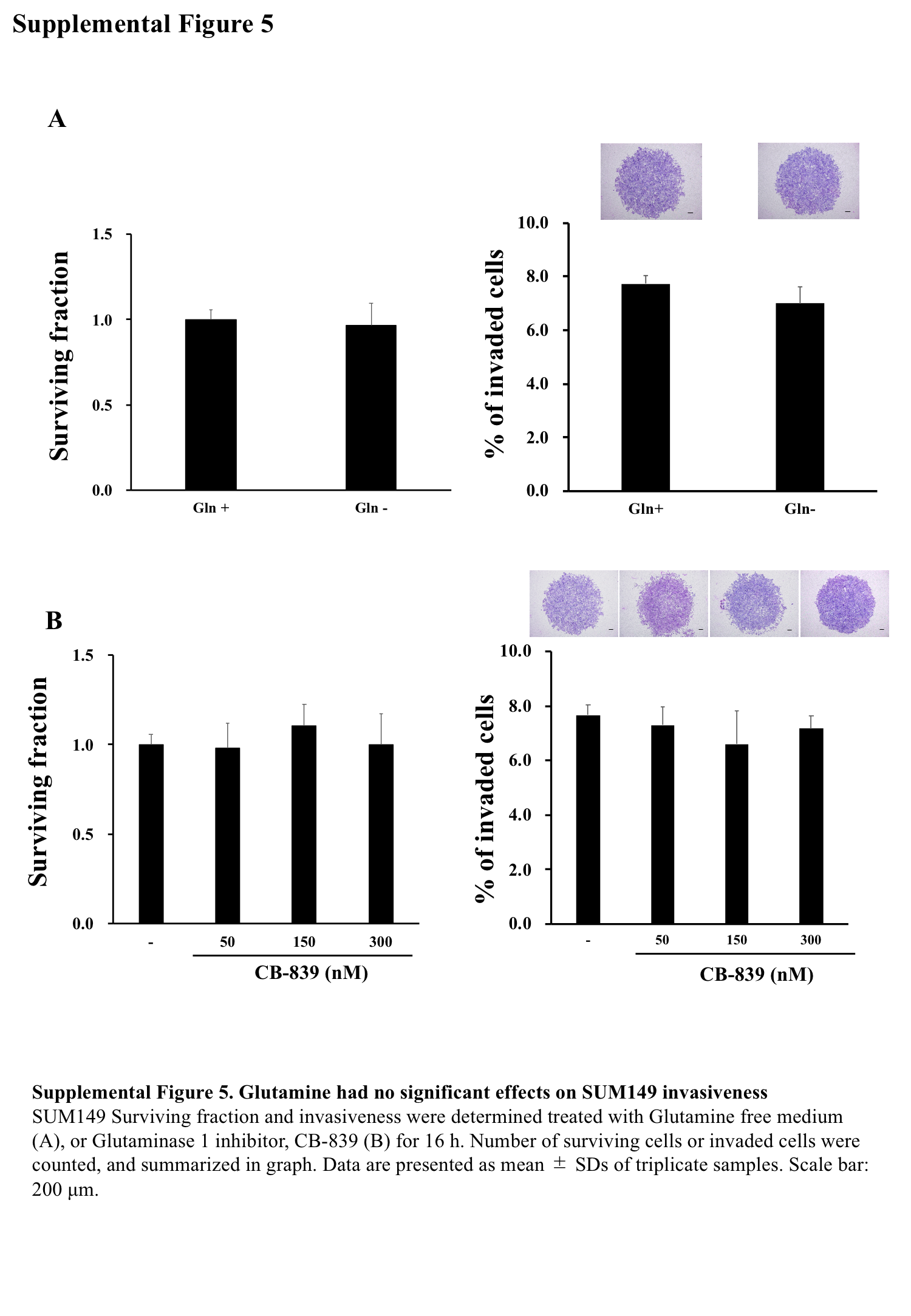

Supplement: Supplementary file 5 — Additional file 5: Supplemental Figure 5. Glutamine had no significant effects on SUM149 invasiveness. [file 12885_2020_7414_MOESM5_ESM.tiff]

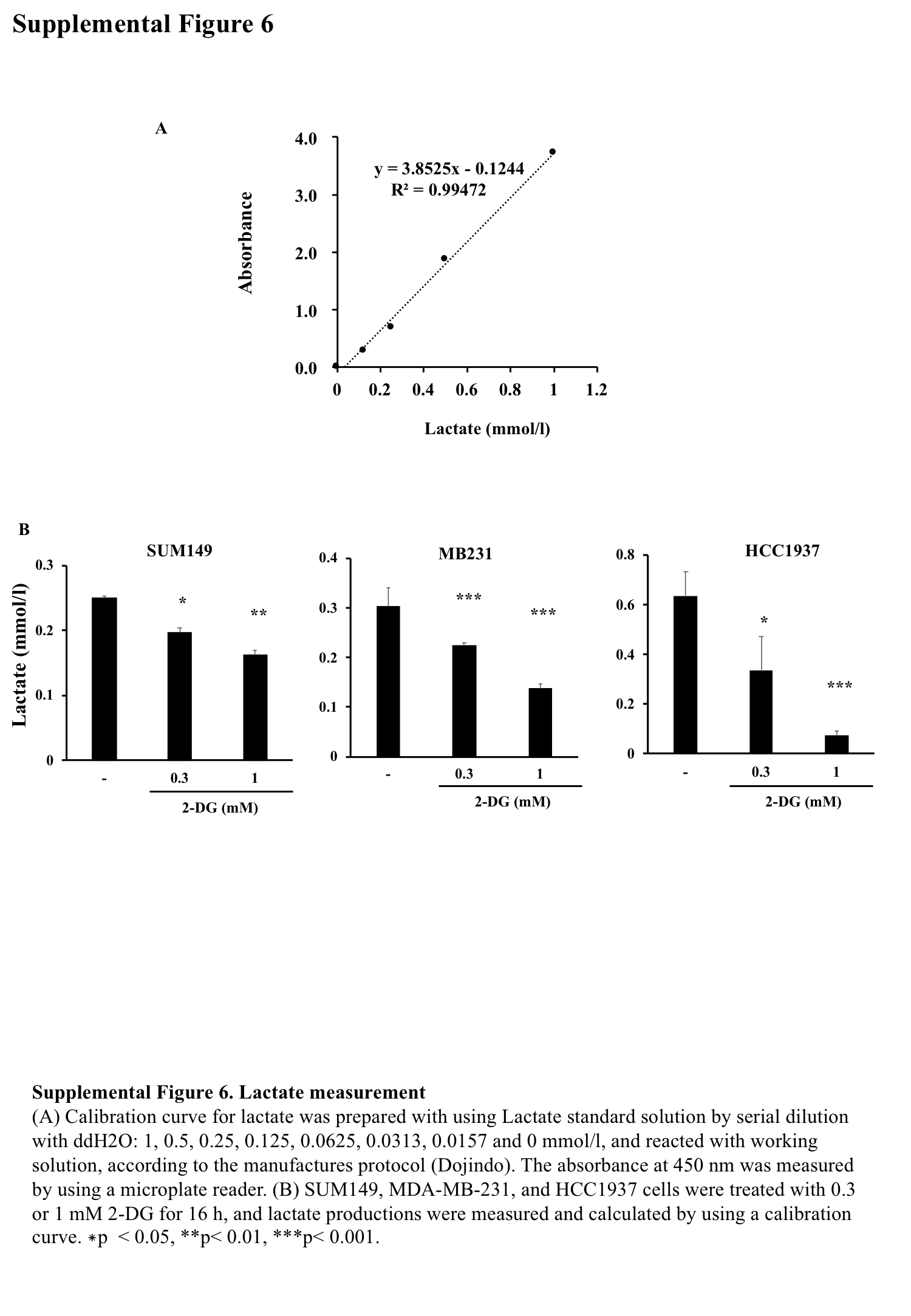

Supplement: Supplementary file 6 — Additional file 6: Supplemental Figure 6. Lactate measurement. [file 12885_2020_7414_MOESM6_ESM.tiff]
